# The accuracy of using guided endodontics in access cavity preparation and the temperature changes of root surface: An in vitro study

**DOI:** 10.1186/s12903-022-02548-w

**Published:** 2022-11-16

**Authors:** Cuifeng Zhang, Xiao Zhao, Cheng Chen, Jingyan Wang, Peiyu Gu, Junchi Ma, Daming Wu, Jin Li

**Affiliations:** 1 Department of Endodontics, Department of Oral & Maxillofacial Imaging, The Affiliated Stomatological Hospital of Nanjing Medical University, 1 Shanghai Road, Nanjing, 210029 China; 2 Department of Stomatology, Women’s Hospital of Nanjing Medical University, Nanjing Maternity and Child Health Care Hospital, Nanjing, 210004 China; 3 Jiangsu Province Key Laboratory of Oral Diseases, 1 Shanghai Road, Nanjing, 210029 China; 4 Jiangsu Province Engineering Research Center of Stomatological Translational Medicine, 1 Shanghai Road, Nanjing, 210029 China; 5 Department of Oral Special Consultation, The Affiliated Stomatological Hospital of Nanjing Medical University, 1 Shanghai Road, Nanjing, 210029 China

**Keywords:** Cone-beam computed tomography, Guided endodontics, Root canal preparation, Temperature

## Abstract

**Background:**

Guided endodontics is a successful technique that has been gradually applied to endodontic therapy in recent years without being affected by the operator’s experience. However, the guided bur produces excessive heat during continuous rotation and friction with root canal walls, it is not clear whether the degree of temperature increase may lead to the periodontal ligament and alveolar bone damage.

**Methods:**

A total of 58 teeth were used, of which 40 teeth were not grouped, all used to evaluate the accuracy. 40 single-rooted premolars were scanned using CBCT and an intra-oral scanner, and 3D-printed guided plates were made with the pre-designed access. A custom-made guided bur was used to prepare the access cavities. The postoperative CBCT data and pre-designed pathways were matched to evaluate the deviation between the planned and virtual paths. The other 18 teeth were randomly divided into three groups (ET20 and ProTaper F3 as the control group, guided endodontics as the test group), with 6 teeth in each group. The temperature changes on the root surfaces were inspected with a thermocouple thermometer.

**Results:**

The average deviation on the tip and the base of the bur was 0.30 mm and 0.28 mm (mesial/distal), and 0.28 mm and 0.25 mm (buccal/lingual). The average angle deviation was 3.62°. The mean root surface temperature rise of the guided endodontics group was the lowest (5.07 °C) (*P* < 0.05).

**Conclusions:**

The access cavity preparation performed with guided endodontics has feasible accuracy and low-temperature rise on the root surfaces. Due to the limitations of the study, whether it has high reliability and safety in clinical applications needs to be further studied in vivo.

## Introduction

Access cavity preparation is the first and crucial step for nonsurgical root canal treatment (RCT) [1]. A properly accessed cavity preparation may achieve a smooth, straight-line path to the apical foramen without changing the original orientation of the root canal, reducing the risk of step formation, zipping, perforation, and separated instruments [2, 3]. Traditional access cavity is mainly based on occlusal anatomy. However, as the crown morphology may change due to aging or pathological factors, it is inaccurate to completely rely on occlusal anatomy for access cavity design [4].

In addition, orthodontic treatment, trauma, chronic inflammation, and age processes often cause pulp calcification and canal obliteration [5, 6]. Studies have shown that pulp calcification is an important cause of root canal orifice location failure and root canal perforation [7]. Dental microscopes and ultrasound equipment are often used when treating teeth with pulp calcification [8], but whether the technique is successful or not highly depends on the clinician’s experience. And after spending a lot of energy and time, the excessive loss of tooth structure will inevitably lead to mechanical structure change, eventually leading to root fracture or other adverse conditions [9, 10], which bring challenges to clinicians and patients.

Recently, with the improvement of cone-beam computed tomography (CBCT) and three-dimensional (3D) rapid prototyping manufacturing technology, the technique of guided endodontics has been introduced to the field of endodontic therapy, including access cavity preparation and endodontic surgery [11, 12]. Guided endodontics has high accuracy and is a successful technique without being affected by the operator’s experience when comparing the drilled path to the planned treatment [13]. They help clinicians achieve predictable and safe results, avoid unnecessary removal of tooth tissue or complications, and improve the treatment prognosis [14, 15]. Although the average planning time takes a long, the preparation of the access cavity using the endodontic guides requires only tens of seconds on average [16–18], which provides a good medical experience for clinicians and patients. Guided endodontics may be a promising method for the endodontic or surgical treatment of complex cases.

However, previous studies used different software to design guided plates and measure deviation, and the diameters of the guided burs (0.85 ~ 1.3 mm) were also different, which led to slight changes in accuracy. Therefore, the accuracy of guided endodontic treatment needs to be further verified by a large number of basic experiments. In general, guided burs with a smaller diameter may have smaller deviations, avoiding cutting dentin tissue excessively. In addition, bone tissue is sensitive to temperatures 10 °C higher than body temperature, which may impair microcirculation and connective tissue, and lead to chronic inflammation in periodontal and adjacent bone tissues [19–21]. However, it has not been reported whether the heat generated by the continuous rotation and friction of the root canal wall during the treatment will lead to a change in root surface temperature, and then damage the periodontal tissue and bone tissue.

Therefore, in the first part of this study, digital design and 3D-printed guide plates were used to access cavity and root canal pathways shaping for isolated teeth, and their accuracy was evaluated. In the second part, the root surface temperature changes during 3D-printed guide plates guided RCT were compared with nickel-titanium instruments and ultrasound instruments commonly used in RCT, and their safety was evaluated, to provide a reference for the further clinical development of guided endodontic treatment.

## Materials and methods

### Stage 1: Accuracy measurement

#### Sample preparation

The approval for this study was obtained from the Ethical Committee Department of the Affiliated Stomatological Hospital of Nanjing Medical University (PJ2018–022-001). Mature human single-rooted premolars without endodontics treatment, crown restorations, caries, periapical lesions, root resorption, and fractures were collected. After removing the residual soft and hard tissues, they were randomly fixed in a curved epoxy model and were scanned using a CBCT scanner (NewTom 5G, QR Srl, Verona, Italy) at 110 kV, 3 ~ 9 mA, a field of view of 8 cm × 12 cm, a basic voxel size of 0.30 mm by an experienced radiologist based on the manufacturer’s operating instructions. The CBCT data was stored, reconstructed, and analyzed using NNT 10.0 software (QR Srl, Verona, Italy). The teeth with similar root lengths, and root and canal diameters were selected.

#### Manufacture guided plates

**Fig. 1:**
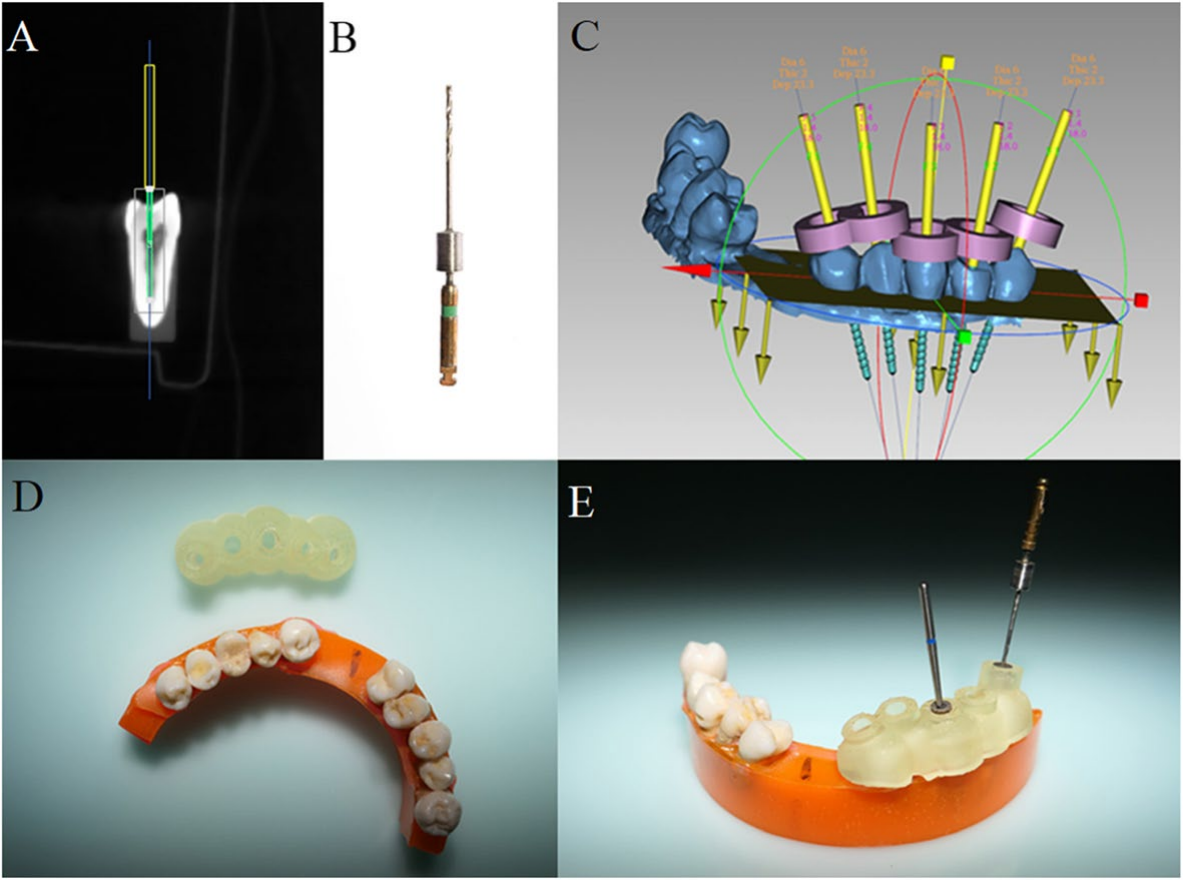
Design and manufacture guided plates. **A**: a virtual bur superimposed to the root canal in the design software; (**B**): the specially designed bur; (**C**): interface of design software for guide plate; (**D**): a top view of the model and guided plate; (**E**): guided plate with metal sleeves positioned on the model, and a specially designed bur and a diamond bur were put in the metallic sleeves

Forty selected premolars were scanned with 3shape Trios intra-oral scanner (TRIOS 3, 3Shape, Denmark). The standard tessellation language (STL) files created by the intra-oral scanner were matched with the CBCT data in a dental implant design software (Digital 3D Implant Sys software, Fox medical tech, China) to design the guided plates. A bur with 0.8 mm diameter and 18 mm work length (Shanghai LZQ Precision Tool, China) was designed for the access cavity preparation using the software and was used to simulate the planned paths (Fig. 1 A-C).

Then the matched data were imported into a 3D printer (Projet 7000MP, 3D System Int, USA), and epoxy resin was used to make 3D-printed guided plates (Fig. 1 D). The guided metal tubes embedded in the plates have a wide inner diameter (3.5 mm) to be compatible with two different sizes of inner sleeves. The inner sleeves are cylindrical tubes with the same outer diameter to match the guided metal tubes. But they have different inner diameters (0.85 mm and 1.4 mm) for the guided diamond bur (1.4 mm diameter) and custom guided bur (0.8 mm diameter), respectively.

#### Access cavity preparation

The 3D-printed guided plates were positioned on the models and their correct and reproducible fitting were examined carefully (Fig. 1 E). A high-speed diamond bur (Komet, Germany) with a maximum diameter of 1.4 mm was used to remove the enamel and the dentin of the pulp chamber. A 10-size K file (21 mm, MANI, INC) was used to check the root canal and to establish the working length. The access cavity was prepared using a special design bur (0.8 mm diameter) at 800 rpm (X-SmartTM, Dentsply Maillefer, Japan) to the apical third of the roots. The bur was cleaned regularly and the canals were irrigated using a 27-gauge needle and 2% sodium hypochlorite during preparation to completely remove dentinal debris. Finally, the instrumented specimens were dried using paper points and scanned again using the CBCT scanner as described above.

#### Accuracy measurement

**Fig. 2:**
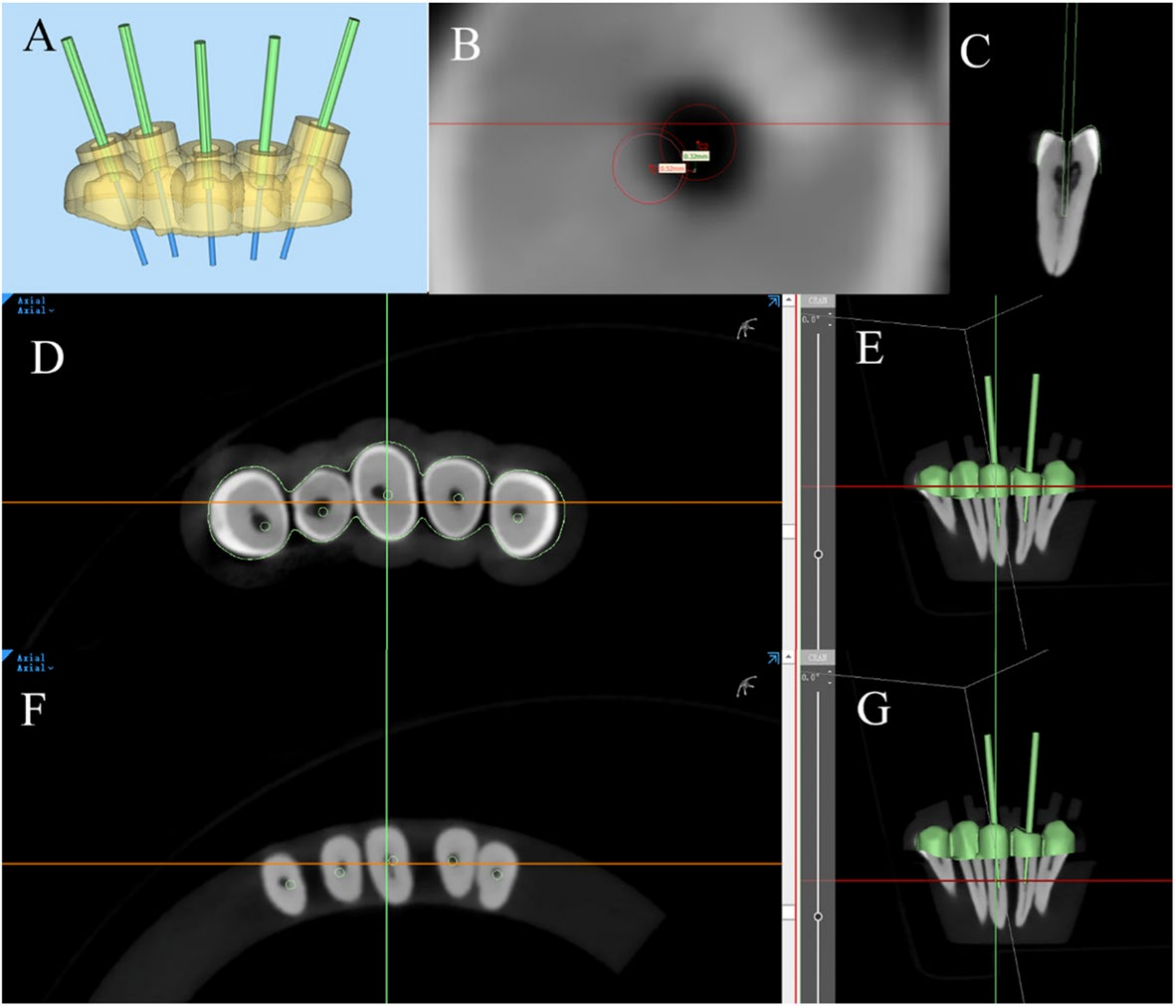
Deviation measurement. **A**: creating virtual bars in Magics software; (**B**): measurement diagram; (**C**): virtual Bar in postoperative CBCT image; (**D**-**E**): the base of the measuring point, red line in E represents the plane of D; (**F**-**G**), the tip of the measuring point, red line in G represents the plane of F

The STL data of the guided rod and the guided plate in the design engineering file was exported and imported into Magics 23.0 software (Materialise NV, Leuven Belgium). A cylinder part with the same height and diameter as the guided bur was created and aligned to the STL data of the guided plate to represent the virtual paths. Then the STL files of the virtual paths and the data of the preoperative tooth surface were imported into Mimics 21.0 software (Materialise NV, Leuven Belgium), and were aligned with the postoperative CBCT image by using a point registration tool (Fig. 2).

The preoperative images were measured in 2 base points, the top point (apical direction) of the Bar as T-Point, and the base point (the entrance near the crown) of the Bar as B-Point. The deviation between the actual T-point and the pre-designed T-point was measured from both buccolingual and mesiodistal directions. Deviation of angle was automatically calculated and output by the Mimics 21.0 software. The part of the artificial identification point was operated by two experienced experimenters independently.

### Stage 2: Temperature measurement

#### Specimen preparation

The access preparation was performed on 18 selected premolars using a high-speed diamond bur. A 10-size K file (21 mm) was used to check the root canals. The canals were prepared with ProTaper nickel-titanium (NiTi) rotary instruments (X-SmartTM, Dentsply Maillefer, Japan) to the middle third of the root length. Then, the specimens were scanned using the CBCT scanner as described above. All CBCT scans were analyzed by NNT software 10.0 at axial planes. The root surface was marked at 1.2 mm thickness of the root canal wall. Then the specimens were divided into 3 groups (*n* = 6): Ultrasonic tip (ET20, Satelec, Pierre Rolland, France), ProTaper F3 file (Dentsply, Ballaigues, Switzerland), and the guided endodontics. The 3D-printed guided plates for the guided endodontics group were made as described above. Standardized bisecting angle digital periapical radiographs were taken of all the teeth from the buccolingual direction using a CCD (Sidexis, Siemens, Germany) system to ensure that the test instruments can directly reach the marked points.

#### Study model

**Fig. 3:**
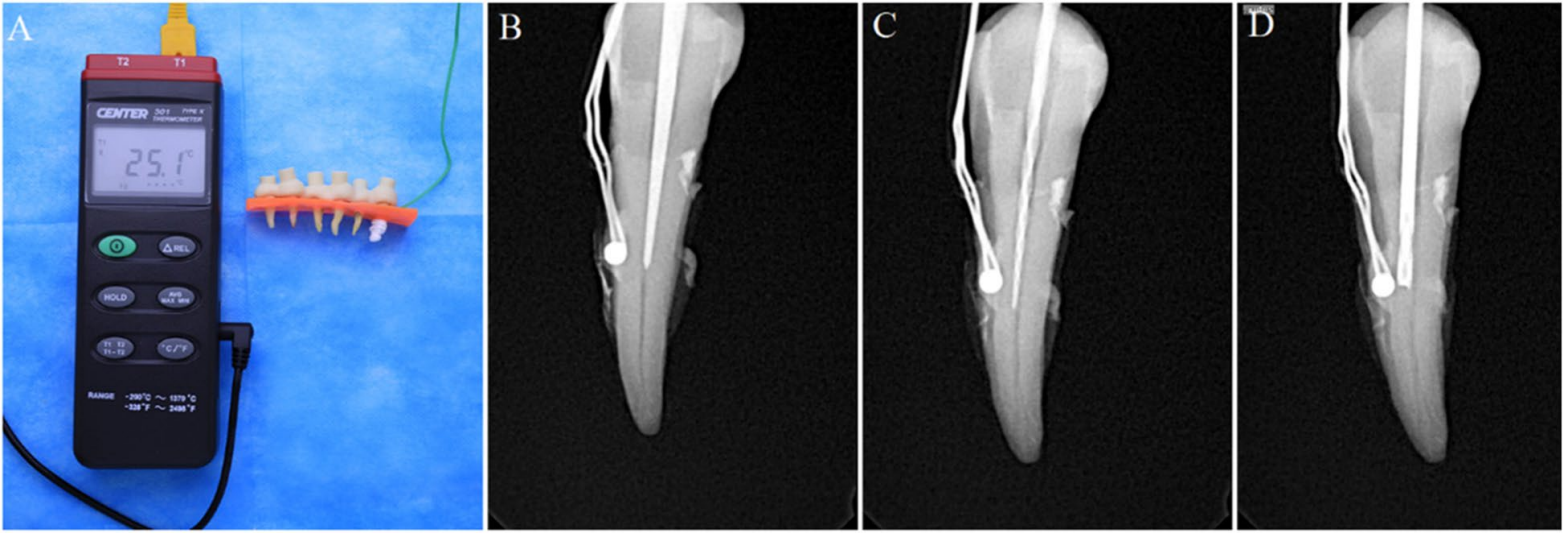
Study model of the temperature measurement and the marked position of the temperature sensor and the test instruments. **A**: study model; (**B**): guided drill; (**C**): F3; (**D**): ET20

A 3 mm thick epoxy resin plate was made to fix onto the cementoenamel junction of the premolars (Fig. 3 A). All roots were completely exposed. A K-type thermometer (Center 301, type-K, tenmars, Taiwan, 0.1 °C) was fixed onto the marked point of the root surface with the polytetrafluoroethylene seal tape to monitor the temperature change according to the manufacturer’s instructions at room temperature (25 °C) (Fig. 3 B-D). Temperature changes were recorded continuously every second but were inspected at 20-second intervals up to 120 seconds.

#### Experimental processes

ET20 group: an ET20 ultrasonic tip was inserted into the canal and reached the marked point of the root. The power level of the ultrasonic device (P5XS, Satelec, Cedex, France) was set at scale 8. The ET20 ultrasonic tip continuously worked for 120 seconds without coolant.

ProTaper F3 group: an F3 file was inserted into the canal and reached the marked point of the root and continuous working for 120 seconds without coolant. A motor and handpiece (X-Smart TM, Dentsply Maillefer, Tochigi, Japan) were used, the speed was set at 800 rpm, and the torque was set at 1.0 N·cm.

Guided endodontics group: guided plate was placed on the models, and the correct and reproducible fitting was examined. The special design bur was inserted into the canal and reached the marked point of the root, and continuous working for 120 seconds as ProTaper F3 group.

The temperature on the root surfaces of all the teeth was recorded automatically using the thermometer.

### Statistical analysis

All data were shown as means ± standard deviation (SD) and analyzed using SPSS 26.0 software (SPSS Inc., Chicago, IL). The 95% confidence interval (CI) of the deviation of the planned and prepared root canal preparation was calculated. The mean temperature rise at the same time between the experimental groups was compared with the T-test. The level of significance was set at *P* < 0.05.

## Results

### Stage 1: Accuracy measurement

Thirty-six teeth successfully reach the working length. Two teeth were excluded due to model dislocation, the other 2 teeth were excluded due to the broken bur. The mean of absolute difference, minimum and maximum deviation at the bur’s tip, and base of the planned and prepared canals in mesial/distal, buccal/lingual (mm) directions, and angle (°) were shown in Table 1.

**Table 1:** The deviation of the planned and prepared access cavity preparation

	Tip of the Bar		Base of the Bar		Angle (°)
	M-D (mm)	B-L (mm)	M-D (mm)	B-L (mm)	
Mean ± SD	0.30 ± 0.20	0.28 ± 0.23	0.28 ± 0.12	0.25 ± 0.20	3.62 ± 1.89
Minimum	0.01	0.01	0.06	0.01	0.23
Maximum	0.75	0.98	0.53	0.79	7.31
95% CI	0.23 **~** 0.36	0.20 **~** 0.36	0.24 **~** 0.32	0.19 **~** 0.32	2.98 **~** 4.26

### Stage 2: Temperature measurement

The temperature rises on the root surface during the 120 seconds of operation were shown in Table 2 (every 20 seconds). The root surface temperature rose gradually and peaked at 120 seconds in all groups. The root surface temperature of the guided endodontics group raised 5.07 °C, which was lower than that of the F3 group (6.58 °C) (*P* = 0.046) and significantly lower than that of the ET20 group (18.17 °C) (*P* < 0.01). There were significant temperature changes between guided endodontics and ET20, guided endodontics, and F3 after 20 seconds (*P* < 0.05).

**Table 2:** The temperature on the root surfaces (Mean ± SD, °C)

Time (second)	ET20	F3	Guided endodontics
0	25.63 ± 0.06	25.60 ± 0.00	25.63 ± 0.06
10	28.60 ± 2.33	27.73 ± 0.40	26.27 ± 0.45
20	31.03 ± 2.76	30.20 ± 1.22	26.90 ± 0.82
30	34.87 ± 0.72	31.80 ± 0.96	27.43 ± 1.04
40	37.70 ± 1.05	32.97 ± 0.80	27.90 ± 1.15
50	39.53 ± 0.96	33.83 ± 0.85	28.23 ± 1.38
60	41.33 ± 0.59	34.50 ± 0.76	28.70 ± 1.49

## Discussion

In recent years, there have been some pre-clinical studies that evaluated the accuracy of guided endodontics and found that its accuracy of it was reliable [22–24]. In this study, the mean absolute difference at the tip of the bur in the mesial/distal direction was 0.30 mm and in the buccal/lingual direction was 0.28 mm, and at the base of the bur was 0.28 mm and 0.25 mm, respectively. The results were consistent with previous studies [22–25], indicating the high precision of the guided endodontics with a negligible effect on the operators and the software for design and measurement. The mean deviation of the angle was 3.62°, which was slightly higher than these previous studies [23, 24]. A possible explanation is that the diameter difference between the metal sleeve inner and the special design bur was small (0.85 mm vs. 0.8 mm), the bur rubbed the metal sleeve inner at high speed, resulting in the slight vibration of the guided plates and thermal deformation of the inner wall of the sleeve, which then led to the deviation of the path. Reducing tolerance between the bur and the slightly oversized sleeve may improve the precision of cavity preparation [22]. In addition, a novel sleeveless 3D-printed guide may be an alternative to the conventional guide design to gain access to obliterated root canals [26].

It is indicated that periodontium would be injured when the temperature raised more than 7 °C and the bone tissue would undergo reversible histologic changes when the temperature raised more than 10 °C for 1 minute [27]. Although dentin is a relatively good insulator, a temperature rise on the external root surfaces with a consequential alveolar bone reaction has been reported after long-term use of warm gutta-percha obturation techniques, NiTi files, retreatment or disinfection with laser, and the operation of ultrasonic instruments [28–31]. For example, Madarati et al [29] measured the temperature rise on the external root surface during the removal of separated NiTi files according to the type of ultrasonic tips, power setting, and contact time, and found that the smaller the tip, the higher the power and the longer the contact time, the higher the temperature rise. The average temperature can rise to 17.5 °C without coolant. Budd et al [31] measured the temperature rise on the root surface caused by ultrasonic post-removal using different devices and techniques and found that the temperature of the root surface raised 12 °C in the 60s and 15.6 °C in the 120 s. There were significant differences in temperature rise as a function of the ultrasonic device, location on the tooth, and cooling method utilized for post-removal.

In this study, the temperature measurement points were set on the external surface of the middle third of the roots with the same dentin thickness confirmed using pre-operation CBCT images. The root surface temperature increased gradually and peaked at 120 seconds in all groups, while the average temperature rise of the guided endodontics was lower than that of F3 and significantly lower than that of ET20. In addition, the root surface temperature of the guided endodontics raised 5.07 °C, which was lower than the safe temperature rise reported in the literature, indicating the safety of the guided endodontics. The ET20 group showed the highest temperature rise and was higher than these in previous studies, which may be related to no coolant during operation, the higher power of the ultrasonic device, and the different thickness of the root canal wall compared with other studies.

It was important to point out that all experimental instruments continuously worked in the air without simulating any heat dissipation in this study. The commonly used cooling measures in clinical include water and intermittent operation. Considering the poor cooling effect of water due to the obstruction of the guide plates in the process of guided endodontics treatment, the experiment was carried out under dry conditions. This kind of continuous work without coolant can’t be applied in clinical practice, for this design was to simulate temperature rise under extreme situations. By observing the temperature change after working for a long time, we can know how long it will be necessary to cool, to provide a reference value for clinical practice. This study shows that it is safe to use this experimental method to guide endodontics therapy even if it works continuously for 120 s under dry conditions. In addition, periodontal blood flow protects the alveolar bone from thermal injury during thermoplasticized root canal obturation [32]. Therefore, it’s reasonable to speculate that the heat generated by access cavity preparation under guided endodontics can be better dissipated by intermittent cutting, cooling with root canal irrigating solutions, and periodontal blood flow.

A limitation of the study is the lack of a sample size calculation, which might be a reason for not finding statistical differences. In addition, a drawback of this in vitro study is the lack of a calcified canal because it was difficult to find enough pulp calcification teeth to test the efficiency of guided endodontic treatment. However, we believe that the guided bur has a strong cutting ability. Secondly, its accuracy is compared with the designed direction and has no absolute correlation with the original direction of the root canal. In addition, the hardness difference between the calcified canal and the surrounding dentin is greater, and the resistance difference between the two sides of the guided bur is greater when the cutting path passes through, which may have some influence on the accuracy. It could be speculated that calcified canal drilling along the designed path may perform at least as well. Of course, The influence of pulp calcification on the efficiency of guided endodontics needs further studies. The application of guided endodontics in calcified root canals has been reported in some clinical cases, the use of guided endodontics in normally calcified teeth enables the preservation of a significant amount of tooth substance, and good therapeutic effects have been achieved [11, 23, 33].

Digitally guided endodontics treatment is a minimally invasive method to deal with pulp calcification or other complex pulp cavity forms in recent years, but no recognized operating standard exists. This study proved a new method for guided endodontics treatment and evaluated whether the heat generated by the continuous operation of this method for a certain period would theoretically cause damage to the periodontal tissue. It can be said that this is a relatively comprehensive in vitro study of guided pulp treatment, which provides a reference for further in vivo research.

## Conclusions

With the limitations of this study, it may be concluded that the access cavity preparation performed with guided endodontics has feasible accuracy and low-temperature rise on the root surfaces, indicating their high reliability and safety in clinical applications in complex RCT.

## Data Availability

The datasets used and analyzed during the current study are available from the corresponding author upon reasonable request.

## Abbreviations

RCT: Root canal treatment
CBCT: Cone-beam computed tomography
3D: Three-dimensions
STL: Standard tessellation language
SD: Standard deviation
CI: Confidence interval
M-D: Mesial-distal direction
B-L: Buccal-lingual direction
